# Monte Carlo-Based Error Propagation for a More Reliable Regression Analysis across Specific Rates in Bioprocesses

**DOI:** 10.3390/bioengineering8110160

**Published:** 2021-10-26

**Authors:** Julian Kager, Christoph Herwig

**Affiliations:** 1 Competence Center CHASE GmbH, 4040 Linz, Austria; 2 Research Division Biochemical Engineering, Institute of Chemical, Environmental and Bioscience Engineering, Technische Universität Wien, 1060 Vienna, Austria; 3 Christian Doppler Laboratory for Mechanistic and Physiological Methods for Improved Bioprocesses, Technische Universität Wien, Gumpendorfer Straße 1a, 1060 Vienna, Austria

**Keywords:** generic error propagation, Monte Carlo, rate calculation, regression analysis, bioprocess evaluation, interlinking of multiple methods

## Abstract

During process development, bioprocess data need to be converted into applicable knowledge. Therefore, it is crucial to evaluate the obtained data under the usage of transparent and reliable data reduction and correlation techniques. Within this contribution, we show a generic Monte Carlo error propagation and regression approach applied to two different, industrially relevant cultivation processes. Based on measurement uncertainties, errors for cell-specific growth, uptake, and production rates were determined across an evaluation chain, with interlinked inputs and outputs. These uncertainties were subsequently included in regression analysis to derive the covariance of the regression coefficients and the confidence bounds for prediction. The usefulness of the approach is shown within two case studies, based on the relations across biomass-specific rate control limits to guarantee high productivities in *E. coli*, and low lactate formation in a CHO cell fed-batch could be established. Besides the possibility to determine realistic errors on the evaluated process data, the presented approach helps to differentiate between reliable and unreliable correlations and prevents the wrong interpretations of relations based on uncertain data.

## 1. Introduction

Verified relations between process parameters and cell-specific characteristics is the key knowledge to develop robust and scalable bioprocesses [1]. This knowledge is often based on regression analysis of historical data, where all possible relations are inspected [2]. Besides classical process parameters such as pH, temperature, and dissolved oxygen, scalable biomass-specific reaction rates are included in this analysis both as independent (such as product formation $q_{p}$) and regressor variables (such as growth µ or substrate uptake rates $q_{s}$) [3,4,5,6]. The knowledge of the interdependencies among these reaction rates ease the scale-up, process design, and control.

The calculation of biomass-specific rates is based on different data sources, including online signals and offline measurements and their timely changes [7]. Different approaches can be followed to obtain the targeted rates, which all include a series of evaluation procedures to maintain the information contained in the signals. The use of smoothing and spline fits might be a good way to obtain smooth time derivatives of noisy measurements [8], but their usage is critical with a low number of measurements with the risk of smoothing out important biological events. Due to the low number of measured samples, the finite differences of two subsequent measurements are most commonly used in biotechnology. As displayed by [7,9], the calculation accuracy of the rates is highly dependent on the measurement frequency and the underlying signal-to-noise ratio of the used measurement methodology. This results in a trade-off between laborious high-frequency sampling including smoothing and spline fits or low-frequency sampling and finite differences among the few measurement points.

To circumvent this trade-off, kinetic models can be used [10]. The underlying reaction kinetics are hereby described by a mass balance model with different reaction kinetics, e.g., first-order or Monod terms [11]. By fitting the model to the data, realistic rate trajectories can be deduced by the model dynamics [12] or by employing model-based state estimation techniques [13,14]. Although the approximation of reaction rates based on an underlying model combined with offline and online measurements leads to good results, an appropriate model and knowledge of the internal reaction dynamics are needed, which is often not the case in biotechnological processes.

If no exact reaction kinetics are known, a constant first-order rate between two measurements can be assumed. In this case, the reaction rates can be determined by solving the mass balance, where the state change is described by a general material balance including inputs, conversion, and output terms. By minimizing the error between the balance equation and the included measurement points, the optimal constant rate can be determined for the analyzed time interval, which can include at least two or more measurement points [15].

No matter what calculation approach is followed, the underlying measurements are prone to errors, which propagates throughout all rate evaluation and regression procedures [16]. To propagate these uncertainties through mathematical functions, a calculus-based approximation or a functional approach can be followed [17,18]. The calculus-based approximation propagates the uncertainty by mathematical error propagation laws, whereas the functional approach re-evaluates the function by including the expected or observed ranges of the measurements. Although computationally efficient, the propagation rules for the calculus approach need to be derived specifically for every evaluation procedure, which is not straightforward for more sophisticated functions such as least squares regressions or differential equation solvers [17]. Due to its easier implementation and generic applicability, especially in numerical- and spreadsheet-based evaluation software, the functional approach is often preferred [18]. By re-evaluating the function with the highest measurement deviations, upper and lower confidence bounds on the results can be determined. As in practice, measurement errors occur randomly, the mentioned procedure potentially overestimates the propagated errors and gives rather a realistic error estimate, a worst-case scenario that hinders the interpretability of the calculated outputs, as discussed by [9] for biotechnological processes.

With today’s computational power, Monte Carlo sampling approaches are gaining more and more attention [19]. This consists of repeating the calculations by varying the input randomly within the stated limits of precision [20]. According to [21], error propagation based on Monte Carlo sampling is the most reliable approach to assign realistic errors to calculated results. For biotechnological processes, Monte Carlo methods have already been successfully used to determine rate calculation errors [13], confidence bounds of model parameters [22], and simulations outputs [23]. The determination of the realistic uncertainties of the target variable is of central relevance for further correlation and regression analysis, where visual inspection model identification and process design can be significantly facilitated by the inclusion of measurement and calculation uncertainties. For specific reaction rates, uncertainties in the range of 20% have been reported to be suitable for conclusive interpretation [13] and process control [24].

Bivariate and multivariate regression are hereby a standard analysis to identify and describe input and output dependencies. Although weighted least squares regression is able to include errors on the predictor variables, possible errors on the regressor variables are often not considered [25]. York (1966) [26] introduced an algorithm that enabled linear regression for data with errors in both the regressor and predictor variables. In addition to finding the best fitting parameters in the case of imperfect measurements, some other important outputs of regression analysis are the parameter and prediction confidence intervals, giving information on the reliability of the found relations. According to [27], Monte Carlo sampling is also well suited to evaluate, uncertainty in regression analysis, which was shown by [21] for geochronology and by [22] to determine the parameter confidence intervals of a nonlinear biotechnological model.

For targeted process development, efficient process transfer, and the definition of operational spaces, it is important to deduce reliable and transferable information. Within this contribution, we show therefore a generic Monte Carlo error propagation approach to obtain a realistic error estimate on both the regressor and predictor variables based on real measurement errors and how to use them, to determine uncertainty in subsequent regression analysis. Based on the known uncertainty of the target variables, the most suitable one can be selected for subsequent regression, and expected impacts can be determined. This greatly facilitates the right conclusions and expectations, leading to a quicker process development and time to market.

The paper is organized as follows: The determination of the scalable reaction and their errors determined by Monte Carlo sampling are described in Section 2. In the subsequent Section 3, the propagated errors are included in the regression analysis, resulting in trustable confidence bounds for the parameters and predictions. Based on these regression models, effective control limits for an *E. coli* and a CHO fed-batch process were established. After discussing the relevance of the obtained results (Section 4), the contribution concludes with a strong suggestion to include measurement errors wherever possible (Section 5), which is strongly facilitated by Monte Carlo sampling procedures.

## 2. Materials and Methods

### 2.1. Experimental Data and Measurements

Data from *Escherichia coli* were obtained from 4 fed-batch cultivations executed in a parallel reactor system (DasGip Force, Eppendorf GmbH). The Fab-fragment-producing *E. coli* strain was cultivated on defined media given in [28], and different exponential glycerol feed profiles were applied during growth, whereas the inducing lactose feed was kept the same for all experiments. Temperature was kept at 37 °C and pH 6.8 by the addition of 12.5% NH3, which was consumed as the main nitrogen source. Dissolved oxygen was kept over 30% by the addition of pure oxygen to the air inflow. Total flow was kept at 2 vvm and the stirrer speed at 1200 rpm. Samples were taken after the batch end, right before induction, and every 2 h during induction. Cells were separated from the liquid (4500 rpm at 4 °C), and after washing the cell pellet with RO water, the pellet was dried at 100 °C to determine the cell dry mass after reaching a constant weight. An adequate dilution of the supernatant was analyzed by HPLC (refractive index), and from a filtrate of homogenized (high-pressure homogenizer) cells, the product amount was quantified by HPLC (UV); more details can be found in [29].

An industrial CHO cell line was cultivated in a chemically defined medium; see [30]. The fed-batch process was carried out in a 3.6 L bioreactor system (Labfors 5, Infors, Switzerland) with an initial working volume of 2 L. The closed-loop controlled process parameters were the temperature (37 °C), the pH value (6.8), the dissolved oxygen tension (40%), and the partial pressure of carbon dioxide (125 mbar). The experiment was performed using three different feeds, namely a glucose feed, a glutamine feed, and a feed with other potentially limiting components. Samples were taken once a day. Total and dead cell counts were determined by an automatic cell counter (Cedex Hi res, Roche). The concentrations of media components were analyzed by an enzymatic analyzer (Cedex Bio HT, Roche).

### 2.2. Measurement Errors

An overview of the measurement errors and their origins is given in Table 1. If available, the measurement accuracies of the analytical devices were taken from the device specification (manufacturer). To determine the individual measurement uncertainties, triplicates were measured for dry cell weights and HPLC measurements and duplicates for cell counts. To evaluate the accuracy of the product analysis of the used *E. coli* strain, the measurements were repeated four times for one specific sample, including homogenization, inclusion body solubilization, and rebuffering, as described in [29].

### 2.3. Rate Calculation

The biomass specific rates were calculated under the usage of a simple material balance equation for the corresponding component, as suggested by [15]. Hereby, biomass-$c_{X}$-specific growth μ and production $q_{p}$ rates can be described by considering dilution $D=\frac{F_{i}n}{V}$ and the timely change in concentration $\frac{dc_{i}}{dt}$ according to Equation (1).

(1) $$\begin{array}{ccccc} \frac{dc_{X}}{dt} & \; & = & \; & \mu \;c_{X}-D\;c_{X}\\ \frac{dc_{P}}{dt} & \; & = & \; & q_{P}\;c_{X}-D\;c_{X} \end{array}$$

For substrate uptake rates, an additional substrate feed ($F_{S}$) with the concentration ($c_{S,feed}$) was considered in the mass balance, given in Equation (2).

(2) $$\frac{dc_{S}}{dt}=q_{S}\;c_{X}+\frac{c_{S,feed}\;F_{S}}{V}-D\;c_{X}$$

Biomass-specific rates (e.g., $q_{S}$) were determined using Nelder–Mead optimization (MATLAB 2020a:fmin), where the ordinary differential equation (MATLAB 2020a: ode23) was iteratively solved between each measurement interval ($t\in \left[t_{k-1},t_{k}\right]$). The optimal rate was identified at the minimum distance between the mass balance equation result (e.g., $c_{S,t}$) and included measurements (e.g., $c_{S,t,meas}$). In comparison to the calculations using the finite differences [7], this calculation allows including the feed and dilution dynamics between the evaluated measurement points and ensures closing overall mass balances.

(3) $$\begin{array}{cc} \; & \min_{q_{S}}{\left(c_{S,t}-c_{S,t,meas}\right)}^{2}\\ \mathrm{subject}\mathrm{to}\\ \; & \frac{dc_{S}}{dt}=q_{S}\;c_{X}+\frac{c_{S,feed}\;F_{S}}{V}-D\;c_{X}\\ \; & t\in \left[t_{k-1},t_{k}\right] \end{array}$$

### 2.4. Linear Least Squares with Errors in Both Variables

The least squares approach is a widely used method to fit an equation to the data. This is performed by minimizing min(S) the sum of squares of the residuals ${\left(\bar{Y}-\bar{y}\right)}^{2}$, where $\bar{y}$ is the measured and $\bar{Y}$ the predicted output. Weighted least squares can be used for problems that are subject to uncertainties in the predictor variables by adding a weighting $W_{y}$, as shown in Equation (4). The second term of the displayed objective includes also the residuals of the independent variables ${\left(\bar{x}-\bar{X}\right)}^{2}$ weighted by $W_{x}$.

(4) $$S=\sum_{i=1}^{n}\left[W_{y,i}\;{\left(\bar{y_{i}}-\bar{Y_{i}}\right)}^{2}+W_{x,i}\;{\left(\bar{x_{i}}-\bar{X_{i}}\right)}^{2}\right]$$

York (1966) [26] developed a widely used procedure to efficiently solve the displayed objective including error weighting for the predictor and independent variables for a straight line fit. The weights $W_{y,i}$ and $W_{x,i}$ are defined as the reciprocal of the variance of the measurement, $W_{\mathrm{ii}}=\frac{1}{\sigma_{i}^{2}}$. A detailed description and comparison to other available algorithms can be found in [25].

### 2.5. Monte Carlo Sampling for Error Propagation and Regression Analysis

For error propagation, rate calculations were repeated 500 times by a Gaussian sampling procedure from the input uncertainty, described in Algorithm 1. From the obtained results, the time-dependent standard error ($\bar{\sigma }$) was calculated. The represented relative error (Figure 2) is the average standard deviation, normalized by the correspondent value of the variable. To propagate the error through multiple calculation steps (indicated as function *f*), the 500 results ${\bar{y}}_{i-1}$ from the previous steps were used as the inputs for the subsequent calculation steps *i* and iterated N times (N = 500) with Gaussian distribution (R∼N(0,1) and with the standard deviation ${\bar{\sigma }}_{i-1}$ of the function inputs ${\bar{y}}_{i-1}$), resulting in the final output uncertainty $\bar{\sigma_{i}}$.
**Algorithm 1** Monte Carlo sampling for error propagation chain.**Calculation Step 1****for**k=1:N**do**${\bar{x}}_{\mathrm{sampled}}=\bar{x}+\bar{\sigma }\;;R∼N\left(0,1\right)$$\bar{y_{k}}=f\left({\bar{x}}_{\mathrm{sampled}},t,\theta \right)$**end for**${\bar{y}}_{1}=\frac{\sum_{k=1}^{N}{\bar{y}}_{k}}{N}$${\bar{\sigma }}_{1}=\sqrt{\frac{\sum_{k=1}^{N}({\bar{y}}_{k}-\bar{y}{)}^{2}}{N-1}}$**Calculation step i****for**k=1:N**do**${\bar{y}}_{i,\mathrm{sampled}}={\bar{y}}_{i-1}+{\bar{\sigma }}_{i-1}\;;R∼N\left(0,1\right)$$\bar{y_{k}}=f\left({\bar{y}}_{i,\mathrm{sampled}},t,\theta \right)$**end for**${\bar{y}}_{i}=\frac{\sum_{k=1}^{N}{\bar{y}}_{k}}{N}$${\bar{\sigma }}_{i}=\sqrt{\frac{\sum_{k=1}^{N}({\bar{y}}_{k}-\bar{y}{)}^{2}}{N-1}}$

Similar to the error propagation, a Monte Carlo sampling procedure was used for the regression analysis. As described in Algorithm 2, the regression was calculated N times (N = 500) under the consideration of the Gaussian distribution ($\bar{\sigma }\;R∼N\left(0,1\right)$), the deflection of independent ${\bar{x}}_{\mathrm{sampled}}$, and predictor ${\bar{y}}_{\mathrm{sampled}}$ variables. From the N obtained parameter sets $P_{k(1:N)}$, the parameter covariance ${\mathrm{cov}}_{P}$, parameter uncertainty (${\bar{\sigma }}_{P}$), and prediction uncertainty ${\bar{\sigma }}_{\hat{y}}$ can be calculated according to the equations given in Algorithm 2.
**Algorithm 2** Monte Carlo sampling for regression analysis.**for**k=1:N**do**${\bar{x}}_{\mathrm{sampled}}=\bar{x}+{\bar{\sigma }}_{x}\;;R∼N\left(0,1\right)$${\bar{y}}_{\mathrm{sampled}}=\bar{y}+{\bar{\sigma }}_{y}\;;R∼N\left(0,1\right)$$\hat{P_{k}}=\underset{P_{k}}{arg min}\;S\left(P_{k}\right),$${\hat{y}}_{k}=f\left({\bar{x}}_{\mathrm{sampled}},\hat{P_{k}}\right)$**end for**$cov_{P}=cov\left(P_{k(1:N)}\right)$${\bar{\sigma }}_{P}=\sqrt{\mathrm{diag}(cov_{P)}}$${\bar{\sigma }}_{\hat{y}}=\frac{\sum_{k=1}^{N}({\hat{y}}_{k}-\bar{y}{)}^{2}}{N-1}$

Assuming a Gaussian distribution, the obtained standard deviations of the rate evaluation ($\bar{\sigma }$), as well as standard deviations from regression analysis including the parameter deviation (${\bar{\sigma }}_{P}$) and the standard deviation of the prediction (${\bar{\sigma }}_{\hat{y}}$) covered 68.3% of the population. For the confidence bounds, 1σ was used, whereas for the control limit, 3σ (99%) was used.

## 3. Results

### 3.1. Propagation of the Analytical Uncertainties in the Data Evaluation Procedures

**Figure 1:**
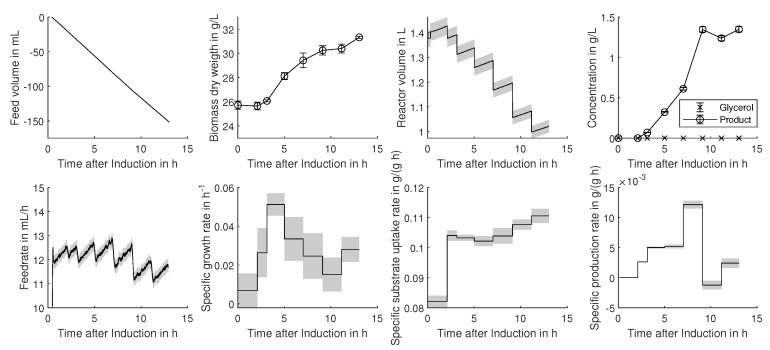
Time-resolved input and rate calculation output data for an exemplary cultivation (*E. coli*). The rate calculation results are displayed as the black line, and the associated uncertainty, obtained by Monte Carlo resampling, is displayed as grey shading.

**Figure 2:**
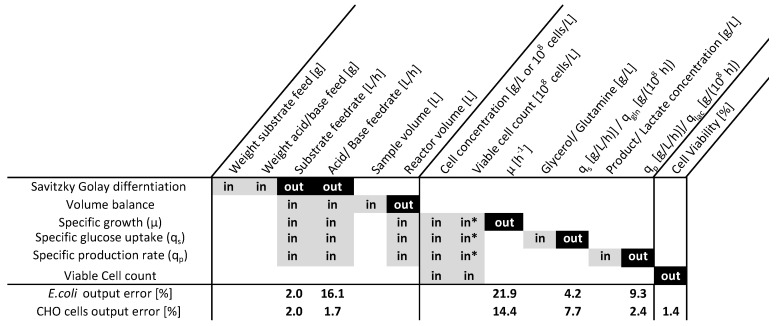
Rate calculation procedure for *E. coli* and CHO cell cultivation datasets, presenting the inputs (in) and outputs (out) of the single calculation steps and the relative average error for the outputs obtained by a Monte Carlo error propagation. The target variables for the subsequent analysis were the specific growth rate (μ), the cell-specific glycerol ($q_{s}$) and glutamine ($q_{Gln}$) uptake rates, the recombinant protein ($q_{p}$) and lactate ($q_{(}Lac)$) formation rates, and the cell viability. * The viable cell count was used as an additional input for the CHO cell process.

Two cultivation datasets were analyzed according to the described procedure. The dependency matrix (Figure 2) visualizes the overall rate calculation procedures indicating the inputs and outputs of the single evaluation steps in a hierarchical order. According to the procedure in Algorithm 1, the output uncertainty (out) was used as the input uncertainty (in) for the next calculation step. Monte Carlo sampling occurred under the consideration of the measurement errors, which are summarized in Table 1. Within Figure 1, the time-resolved measurement inputs and the correspondent output uncertainties are displayed for an analyzed process.

Feed rates were calculated via Savitzky–Golay differentiation (2nd-order polynomial, 50 data points as the window), including balances with an accuracy of ±0.1 g. Error propagation revealed a precision of 2% for substrate feed and only 16% for the high dynamic acid and base feeds. The highest uncertainties can be observed for the calculation of the specific growth rate (μ), which is based on Equation (1) and always solved between two measurement points. Due to the usage of more accurate cell count measurements instead of dry cell weights, the growth rate determined for the CHO cell cultivation was more precise (14.4% uncertainty) compared to the average error for the *E. coli*. Unlike the growth rate, the biomass specific substrate uptake and production rates can be determined with uncertainties below 10%. This can be explained by error propagation laws as the error of biomass is not multiplied as for the calculation of the growth rate.

Within Figure 1 and Figure 2, time-resolved calculation inputs, as well as rate calculation results are displayed for one of the four analyzed *E. coli* processes. As discussed above, a high uncertainty in the growth rate can be seen, although the input measurements showed reasonable low errors. The growth rate is very imprecise, and therefore, it is hard to observe significant changes over time, which can hinder subsequent correlation and regression analysis. Overall, the uncertainty lies within the reported uncertainties of 20% [13,24] and can therefore be regarded as reliable.

### 3.2. Regression Analysis Based on Uncertain Data

Biomass-specific rates offer the possibility to determine scalable and transferable relationships, as well as to discover product formation kinetics and metabolic behavior. In the simplest case, these relations can be described by a straight line, which can be seen for the maximal reached biomass-specific production rate $q_{p}$ and the specific substrate uptake rate $q_{s}$ for the examined *E. coli* processes and for the cell-specific lactate production ($q_{lac}$) and glutamine uptake ($q_{gln}$) for the CHO cell process.

#### 3.2.1. *E. coli*

**Figure 3:**
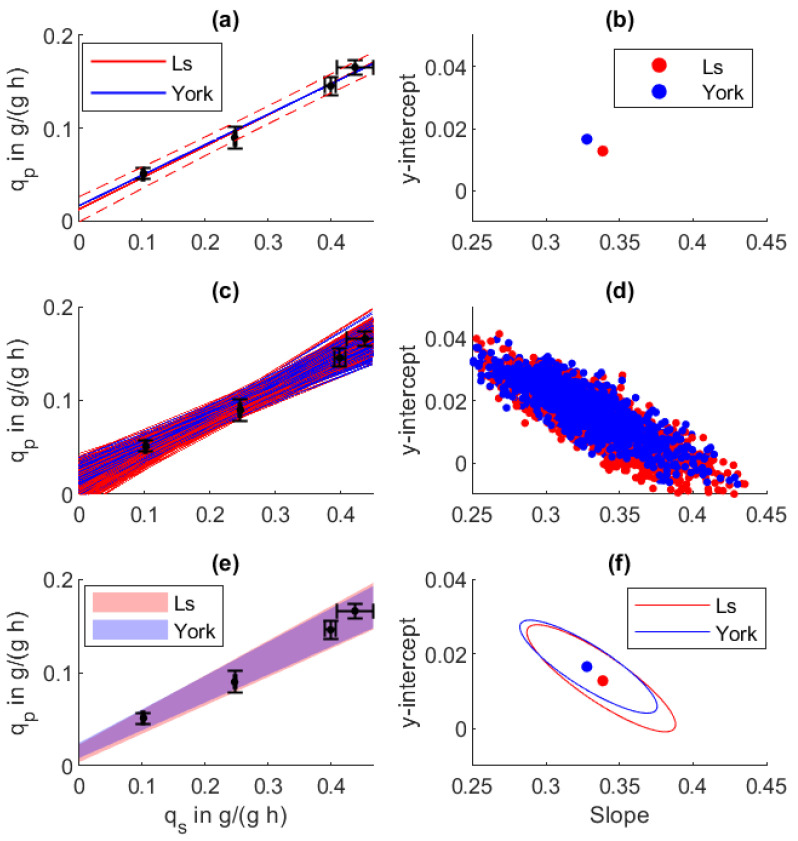
Regression analysis between biomass-specific substrate uptake $q_{s}$ and specific production rate $q_{p}$ in an *E. coli* fed-batch process. (**a**) Regression line and (**b**) regression parameters from normal least squares (LS) and York regression (York). (**c**,**d**) One-thousand Monte Carlo regressions based on the uncertainties of the specific rates. (**e**,**f**) The obtained 68.3% parameter confidence intervals and resulting prediction confidence.

The linear relation between the maximal reached biomass-specific production rate $q_{p}$ and the specific substrate uptake rate $q_{s}$ for the examined *E. coli* is displayed in Figure 3, and the resulting parameters are summarized in Table 2.

In Figure 3a,b, standard linear regression is displayed as Ls. Under the usage of York regression including error weighting based on $q_{s}$ and $q_{p}$, different results for the y-intercept and the slope were obtained, as displayed in Table 2 and summarized in Table 2. As indicated by the error bars, high $q_{s}$ values are less precise. Whereas in a normal or weighted least squares approach, this high uncertainty is ignored, York regression includes this point with a reduced weight on the overall fit.

Single regression results from the Monte Carlo sampling are displayed in Figure 3c,d from which the 68.3% confidence bounds and the error ellipse were calculated, displayed in Figure 3e,f. Besides showing narrower confidence bounds, the York regression indicated an overall higher slope and a negative y-intercept. The error ellipse in (d) shows that under the used data, the y-intercept and the slope were cross-correlated, whereas two local clusters were formed based on the two used regression procedures (f).

Although a positive correlation between $q_{s}$ and $q_{p}$ can be seen, the correct parametrization of a line is hampered by the cross-correlation of the y-intercept and the slope. By integrating the propagated measurement uncertainty under the usage of York regression, the 68.3% error ellipse (Figure 3f) can be significantly narrowed, whereas for the standard least squares (Ls) regression, further data points close to the y-intercept would be necessary to reduce cross-correlation. Within Table 2, the final regression parameters are summarized. As already validated by the original paper [26], the York regression parameters and their error estimate was not affected by the Monte Carlo resampling procedure. The Monte Carlo resampling (Ls MC) revealed high scattering and large confidence bounds under the usage of normal least squares regression. The intercept shows an error of over 400% and the slope 8.9%, whereas the uncertainties from York regression are much smaller with 16.6% on the y-intercept and 2.3% on the slope.

#### 3.2.2. CHO Cells

**Figure 4:**
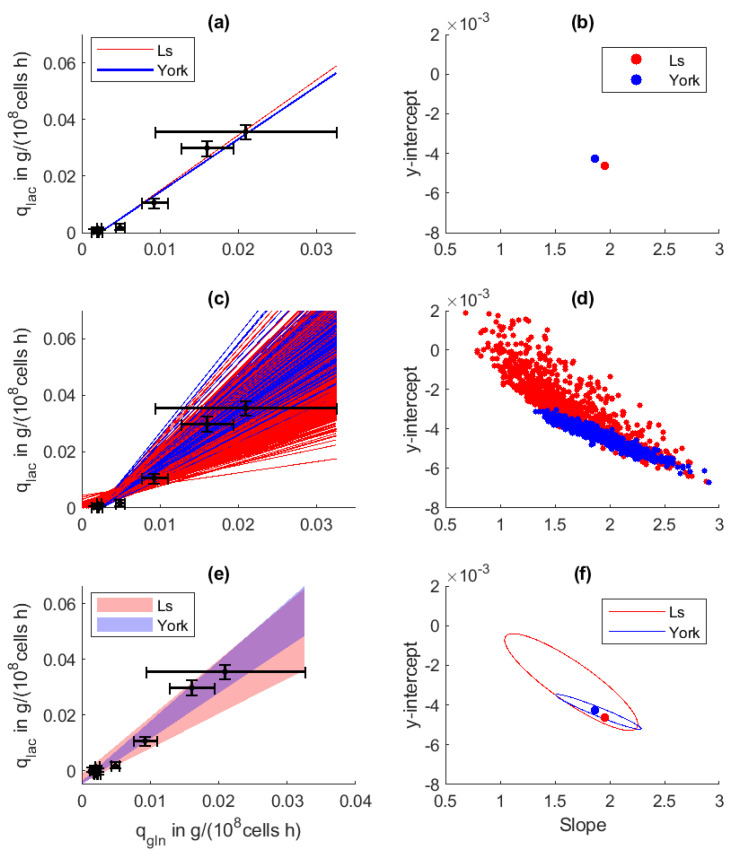
Regression analysis between cell-specific glutamine uptake $q_{gln}$ and specific lactate formation rate $q_{lac}$ in a CHO cell fed-batch process. (**a**) Regression line and (**b**) regression parameters from normal least squares (LS) and York regression. (**c**,**d**) One-thousand Monte Carlo regressions based on the uncertainty of the specific rates. (**e**,**f**) The obtained 68.3% confidence intervals on the regression line and the regression parameters.

For the CHO cell process, the relation between glutamine uptake $q_{gln}$ and lactate production $q_{lac}$ was investigated. The respective linear regressions are shown in Figure 4, and the resulting parameters are summarized in Table 3. In Figure 4a,b, the standard linear regression is displayed as Ls. Under the usage of York regression, propagated errors on $q_{gln}$ and $q_{lac}$ were included in regression analysis. Although the regression lines and parameters in Figure 4a,b are well aligned, the subsequent Monte Carlo procedure revealed the importance of York’s error weighting. Due to the high uncertainty in high glutamine uptake calculations, the normal Ls procedure is strongly influenced by this high error, leading to high scattering in the regression parameters (Figure 4d and Table 3 Ls MC). In Figure 4e,f, the resulting 68.3% confidence bounds are shown for the examined CHO cell process. Similar to the *E. coli* example, the confidence bounds especially of the regression parameters significantly decreased under the usage of error weighting.

The Monte Carlo sampling revealed 57.1% error on the y-intercept and 24.5% on the slope resulting from the LS regression procedure, whereas York regression yielded only 14% error on the slope and the y-intercept. Both methods resulted in a negative y-intercept, which indicates potential lactose metabolization at low glutamine availability. A closer view of Figure 4d reveals that due to the high uncertainty of the determined $q_{gln}$, the y-intercept of the normal least squares regression also reaches positive values. In this regard, a conclusive statement is hindered.

### 3.3. Determination of Confidence Bounds for Control

**Figure 5:**
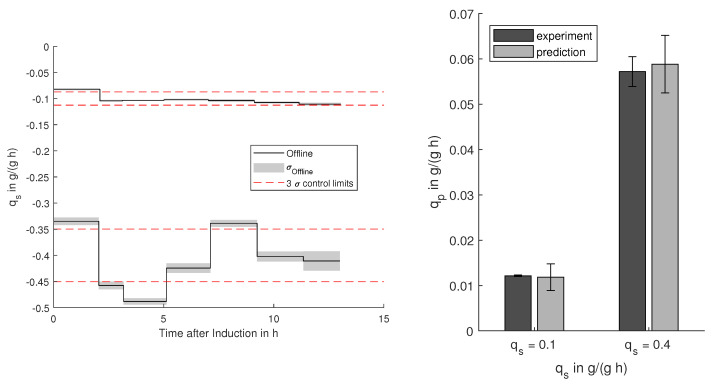
Control limits for high (−0.4 g/(g h)) and low (−0.1 g/(g h)) biomass-specific substrate uptake ($q_{s}$) during induction with the offline-determined uptake rates and their standard deviation $\sigma_{\mathrm{offline}}$. Resulting and predicted productivities $q_{p}$ of the two processes with the 99% prediction confidence and standard deviation for the measured productivities.

**Figure 6:**
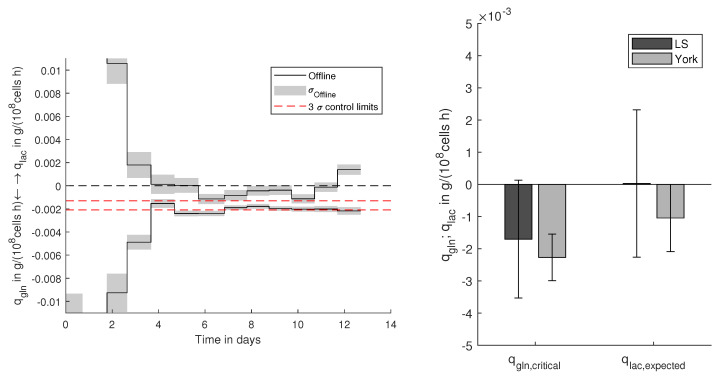
Control limits for cell-specific glutamine uptake ($q_{gln}$) to avoid lactate ($q_{lac}$) production with offline-determined specific rates with their standard deviation ($\sigma_{\mathrm{offline}}$) of the examined CHO cell process. Deduction of control limits based on York and LS regression with their expected lactate production with 99% confidence.

For production processes, the extracted relations between manipulable process and biological parameters are of utmost importance to ensure optimal operation. The production and consumption rates are hereby of central interest to guarantee efficient substrate-to-product conversion. Besides having the possibility to identify relations even with uncertain data sources, controllable limits have to be deduced, to ensure a consistent process outcome. The definition of practicable control limits and the prediction of the expected results is a challenging task and is highly dependent on the expected and present uncertainties. On the one hand, if the control limits are too narrow, there is a high risk of discarding batches based on random and unavoidable errors. On the other hand, generous control limits are not effective to detect critical deviations. Based on the propagated and predicted uncertainties, the best trade-off and reliable control limits, as well as expected output ranges can be defined as displayed in Figure 5 for the *E. coli* and in Figure 6 for the CHO cell process. Hereby, the realistic uncertainties on the relations between the target variables are considered and offer the determination of statistically sound and effective control limits.

#### 3.3.1. *E. coli*

For the investigated *E. coli* process, high production rates $q_{p}$ are desirable, which can be reached with high glycerol supply $q_{s}$. In Figure 5, the specific substrate uptake rates during the induction for two processes are shown including the predicted and the obtained productivities $q_{p}$. Based on the propagated uncertainty on the determination of the biomass-specific uptake rate $q_{s}$, 3 σ (99%) control limits can be defined. A working controller is able to keep the value within these limits, whereas fluctuations within these limits are mostly due to propagated measurement uncertainties. For the process with low specific glucose uptake, the control was keeping the set-point within the reachable limits, whereas the higher set-point (0.4 g/g/h) showed slight deviations. With respect to the predicted process outcome, both processes yielded productivities within the predicted limits.

#### 3.3.2. CHO Cells

Lactate can have inhibiting effects on CHO cells. Therefore, the lactate concentration needs to be kept under control and as low as possible during cultivation processes. As shown in Figure 4a–f, glutamine availability enhances lactate production. Since glutamine is an essential component, it needs to be provided to the culture. Based on the retrieved correlation, glutamine supply without a net production of lactate can be defined. As already shown for the previous example, reliable control limits can be defined, which are displayed in Figure 6. Based on the propagated error 3σ (99%), control limits to avoid lactate formation can be defined. For the examined CHO cell process, it can be observed that as long the glutamine uptake $q_{gln}$ is within the control limits, lactate formation $q_{l}ac$ remains negative, indicating slight lactate assimilation and effective prevention of lactate accumulation. Within the bar chart in Figure 6, the deduction of the control limits is shown. Based on the York regression, effective control limits ($q_{gln,critical}$) to avoid lactate production $q_{lac,expected}$ can be defined. As the normal least squares (Ls) yields highly biased regression parameters, no feasible $q_{gln,critical}$ can be defined, so that the 99% confidence bounds would also reach positive $q_{lac,expected}$. Besides that, the expected lactate production is highly uncertain with broad confidence bounds reaching from relatively high net production to high assimilation rates.

### 3.4. Prediction of Harvest Time Point Probability

**Figure 7:**
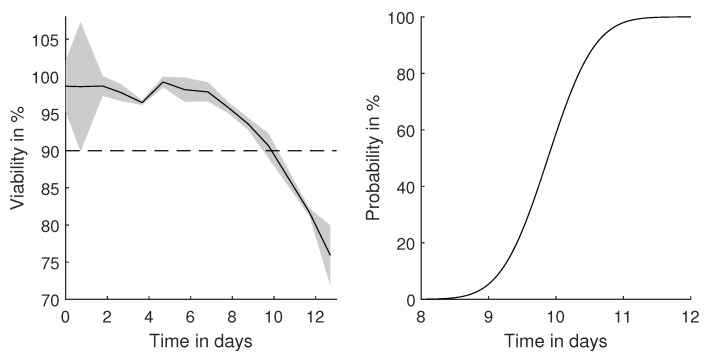
Determined CHO cell viability throughout a fed-batch experiment with a harvest threshold of 90% viability. Based on the propagated uncertainty, the probability of crossing the threshold can be determined.

To deliver a consistent matrix structure of the cell broth for further purification and polishing steps, the harvest time point is a critical process parameter, which can change from batch to batch. For CHO cell processes, commonly, the overall viability is taken as an indicator for the optimal time point of harvest. Although within Table 1, the displayed error on the cell viability (1.4%) is very low, it can still have an impact on the determination of the optimal harvest time point, as displayed in Figure 7 for the analyzed CHO cell process.

Hereby, an exemplary threshold of 90% was selected, and the probability of crossing this threshold was calculated based on the measurement precision. Reaching 90% viability after a few days of cultivation is a typical value for mammalian cultivations [31]. With an exact measurement or by ignoring the measurement error, Day 10 would be the optimal harvest time point. By considering the uncertainty of the determination of the cell viability, a harvest at Day 9 would ensure 90% certainty to be above the selected threshold.

## 4. Discussion

### 4.1. Realistic Quantification of Errors on Determined Specific Rates

As displayed in Figure 2 and within Figure 1, the proposed Monte Carlo procedure offers the possibility to determine realistic uncertainties on evaluated data. This is especially important for the determination of uncertainties on specific rates, as shown by [7]. Although specific rates offer the possibility to deduce transferable and scalable knowledge from experiments, they are subject to high errors, which is important to consider before drawing any conclusion or using them for subsequent root cause analysis, as proposed by [32].

The specific growth rate is an important and widespread variable used as an input for analysis [33] and as a control variable [24]. Since the error propagation revealed that the growth rate is subject to high uncertainties of approximately 20%, further analysis and control should possibly be based on alternative rates that can be determined more precisely, as for example the specific substrate uptake rate $q_{s}$, as shown in a previous work [34].

### 4.2. Error Weighting for a Better Identification of the Regression Parameters

Regression analysis is a central tool for simple and multivariate evaluation procedures in biotechnology. Visual data inspection or correlation analysis are hereby used to discover rough patterns and relations. Subsequent regression analysis aims to deduce a mathematical relation for further usage in process design, monitoring, and control. Although advanced multivariate regression techniques are in fashion [35,36,37], simple straight-line fits and MLR procedures are still widespread and well suited for certain problems [38]. No matter what algorithms are used, all procedures rely on the calibration datasets with underlying errors and uncertainties.

The here shown simple and transferable Monte-Carlo-based determination and inclusion of these uncertainties does not need any additional experimental and analytical effort and enables deriving the best-suited regression parameters and their distributions, no matter what regression or data evaluation procedure is used. Although Monte Carlo methods come with extensive computational costs, they can be easily parallelized, making them suitable for multicore computing, which is increasingly integrated in numerical software.

### 4.3. Achievable Control Limits

The accuracy of any controller is determined by its weakest point. In biotechnological processes, the weakest points are often the measurements themselves, which determine the deviation from the aimed set-points. Hereby, the controller can only act within the precision and the accuracy of the underlying measurements [24,34]. This is important for the definition of suitable control limits around the set-points [39]. Within this contribution, it could be shown how achievable and effective the control limits can be for cell-specific rates to obtain a certain productivity and to avoid overflow metabolism in an *E. coli* and CHO cell process. Hereby, the control limits were based on the determined precision of the calculated rates based on the reference measurements, which were propagated along the regression procedure by the proposed Monte Carlo procedure.

### 4.4. Probabilistic Rather Than Case-by-Case Decisions

Events and their timely detection during dynamic cultivation processes are important to guarantee consistent product quality. Their definition and detection is widely discussed within the scientific community [40]. One important event is the at-line monitoring of the cell viability [31,41,42] and other components [43]. As these analytics are subject to errors, a decision based on them can vary from batch to batch. Under the usage probabilistic decisions, consistency can be ensured. Propagated errors are hereby the basis to evaluate the probability to cross predefined thresholds, as exemplarily shown in Figure 7.

## 5. Conclusions

**Figure 8:**
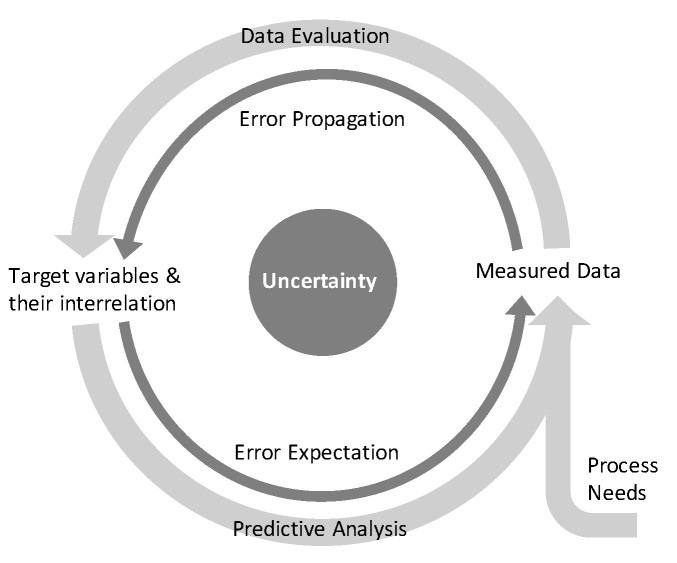
Data evaluation and predictive analysis including uncertainty and propagating it along these procedures.

Within this contribution, a ubiquitous applicable procedure was shown to propagate measurement errors through bioprocess evaluation with the aim to achieve valid correlations between target variables and reliable control limits for manipulable variables, as is schematically displayed in Figure 8. The procedure consists of propagating the crude measurement errors through a series of data evaluation methodologies before both determined errors on regressor and predictor variables were included in a regression analysis. Based on the determined regression uncertainty, expected results and effective control limits can be predicted to meet the process needs.

Based on two industrially relevant organisms, *E. coli* and CHO cells, its applicability to biotechnological cultivations was shown. For the calculation of the cell-specific uptake and production rates, the propagation procedures revealed that with typical sampling frequencies, the specific growth rate μ can be determined with the lowest precision (approximately 20%), whereas the determination of other specific rates showed higher precisions, below 10%. These precisions are important for further regression analysis or for monitoring and control considerations.

Through simple linear regression analysis, correlations between the biomass-specific substrate uptake rate $q_{S}$ and the production rate $q_{P}$ could be determined for *E. coli*, and the relation between the cell-specific glutamine uptake rate $q_{gln}$ and lactate formation $q_{lac}$ for the CHO cell process was determined. Under the usage of these errors, a realistic distribution of the regression parameters, their covariance, and prediction confidence intervals could be determined. Under the usage of error weighting in both the predictor and regressor variables, confidence bounds could be significantly narrowed, without the need for additional data points.

Within three use cases, the usefulness of the error propagation was assessed. For the two examined organisms, control limits could be successfully established to guarantee high production rates in a *E. coli* and to avoid excessive lactate formation in a CHO cell fed-batch. In addition to that, probabilistic decisions were possible, as shown for the harvest time point determination. Based on this, we avoided imperfect measurements being wrongly interpreted, ensuring consistent decisions and the extraction of relevant information, which are important to continuously improve and guarantee the quality of biochemical processes. A sound inclusion of measurement uncertainty and its propagation along process evaluation can additionally lead to a reduction of the needed experimental iterations during process development and enable the assessment of needed measurement accuracies, to obtain the aimed at regression and control accuracies.

## Figures and Tables

**Table 1:** Uncertainties of the measurements.

Raw Signal	Unit	Analytical Device	Error σ	Error Source
Weight substrate feed	g	Balance	±0.1 g	manufacturer
Weight acid/feed feed	g	Balance	±0.1 g	manufacturer
Sampling	mL	Graduated syringe	±3 mL	manufacturer
Cell dry mass	g/L	Drying oven + balance	individual	triplicates
Total cell count	cells/mL	Cedex hi res	individual	duplicates
Viable cell count	cells/mL	Cedex hi res	individual	duplicates
Glycerol	g/L	HPLC (RI)	±3%	triplicates
Glutamine	g/L	Cedex BioHT	±3%	manufacturer
Lactate	g/L	Cedex BioHT	±3%	manufacturer
Product *E. coli*	g/L	HPLC (UV)	±2.5%	repeated measurements

**Table 2:** Summary of the resulting regression parameters of the analyzed *E. coli* fed-batch processes with the standard parameter error for least squares (Ls) and York regression, with and without Monte Carlo (MC) resampling.

Methodology	y-Intercept (g/(gh))	Slope (-)
Ls	0.0003	0.1321
Ls MC	0.0005 ± 0.0021	0.1319 ± 0.0117
York	−0.0038 ± 0.0006	0.1567 ± 0.0036
York MC	−0.0038 ± 0.0006	0.1566 ± 0.0038

**Table 3:** Summary of the resulting regression parameters of the analyzed CHO cell fed-batch process with the standard parameter error for least squares (Ls) and York regression, with and without Monte Carlo (MC) resampling.

Methodology	y-Intercept (g/(10^8^ Cells ∗ h))	Slope (-)
Ls	−0.0046	1.9518
Ls MC	−0.0028 ± 0.0016	1.6480 ± 0.4016
York	−0.0040 ± 0.0005	1.8652 ± 0.2028
York MC	−0.0043 ± 0.0006	1.8965 ± 0.2582

## Data Availability

The underlying data will be made available upon any request.

## Abbreviations

The following abbreviations and symbols are used in this manuscript:

HPLC	High Pressure Liquid Chromatography
CHO	Chinese Hamster Ovary
MC	Monte Carlo
FA	Functional Analysis
Gln	Glutamine
Lac	Lactate
μ	biomass-specific growth rate
$q_{p}$	biomass-specific product formation rate
$q_{S}$	biomass-specific substrate uptake rate
$q_{i}$	biomass-specific rate of component i
$c_{i}$	concentration of component i
*X*	biomass
*S*	substrate
*P*	product
*D*	dilution rate
$c_{S,feed}$	substrate concentration in the feed
$F_{S}$	feed rate
$t_{k}$	t of sample k
$c_{S,t,meas}$	measured concentration at t
$\bar{X}$	true input
$\bar{x}$	reconstructed input by regression
$\bar{Y}$	measured output
$\bar{y}$	predicted regression output
$W_{y}$	weighting matrix of predictions
$W_{x}$	weighting matrix of input variables
*S*	weighted sum of squared error
σ	standard deviation of measured output or true input
*N*	number of Monte Carlo iterations
*f*	arbitrary function converting input $\bar{x}$ to output $\bar{y}$
${\bar{x}}_{\mathrm{sampled}}$	sampled input from Gaussian-distributed error
$\bar{y_{k}}$	calculation result of sampled input
${\bar{y}}_{i,\mathrm{sampled}}$	sampled input from output i
${\bar{\sigma }}_{i}$	standard deviation after calculation step i
$P_{k(1:N)}$	regression parameter for N Monte Carlo evaluations
${\mathrm{cov}}_{P}$	parameter covariance
${\bar{\sigma }}_{P}$	parameter standard deviation
${\bar{\sigma }}_{\hat{y}}$	standard deviation of regression output
